# Plasma unmetabolized folic acid in pregnancy and risk of autistic traits and language impairment in antiseizure medication–exposed children of women with epilepsy

**DOI:** 10.1093/ajcn/nqab436

**Published:** 2022-01-05

**Authors:** Elisabeth Synnøve Nilsen Husebye, Annabel Willemijn Karine Wendel, Nils Erik Gilhus, Bettina Riedel, Marte Helene Bjørk

**Affiliations:** Department of Clinical Medicine, University of Bergen, Bergen, Norway; Department of Neurology, Haukeland University Hospital, Bergen, Norway; Faculty of Medicine, Vrije Universiteit, Amsterdam, The Netherlands; Department of Clinical Medicine, University of Bergen, Bergen, Norway; Department of Neurology, Haukeland University Hospital, Bergen, Norway; Department of Medical Biochemistry and Pharmacology, Haukeland University Hospital, Bergen, Norway; Department of Clinical Science, University of Bergen, Bergen, Norway; Department of Clinical Medicine, University of Bergen, Bergen, Norway; Department of Neurology, Haukeland University Hospital, Bergen, Norway

**Keywords:** anticonvulsants, neurodevelopment, autism spectrum disorder, language delay, folic acid, MoBa, MBRN

## Abstract

**Background:**

Fetal exposure to unmetabolized folic acid (UMFA) during pregnancy may be associated with adverse neurodevelopment. Antiseizure medication (ASM) may interact with folate metabolism. Women with epilepsy using ASM are often recommended high-dose folic acid supplement use during pregnancy.

**Objectives:**

The aim was to determine the association between UMFA concentrations in pregnant women with epilepsy using ASM and risk of autistic traits or language impairment in their children aged 1.5–8 y.

**Methods:**

We included children of women with epilepsy using ASM and with plasma UMFA measurement enrolled in the Norwegian Mother, Father, and Child Cohort Study (MoBa). Data on ASM use, folic acid supplement use, autistic traits, and language impairment were obtained from parent-reported questionnaires during pregnancy and when the child was 1.5, 3, 5, and 8 y old. Plasma UMFA concentrations were measured during gestational weeks 17–19.

**Results:**

A total of 227 ASM-exposed children of 203 women with epilepsy were included. Response rates at ages 1.5, 3, 5, and 8 y were 67% (*n* = 151), 54% (*n* = 122), 36% (*n* = 82), and 37% (*n* = 85), respectively. For 208 (94%) children, the mother reported intake of folic acid supplement. There was no association between UMFA concentrations and autistic traits score in the adjusted multiple regression analyses at age 3 y (unstandardized B: −0.01; 95% CI: −0.03, 0.004) or 8 y (unstandardized B: 0.01; 95% CI: −0.02, 0.03). Children exposed to UMFA had no increased risk of autistic traits at age 3 y [adjusted OR (aOR): 0.98; 95% CI: 0.2, 4.2] or 8 y (aOR: 0.1; 95% CI: 0.01, 1.4) compared with unexposed children. We found no association between UMFA concentrations and language impairment in children aged 1.5–8 y.

**Conclusions:**

Our findings do not support any adverse neurodevelopmental effects of UMFA exposure in utero in children of women with epilepsy using ASM.

## Graphical Abstract

**Graphical Abstract:**
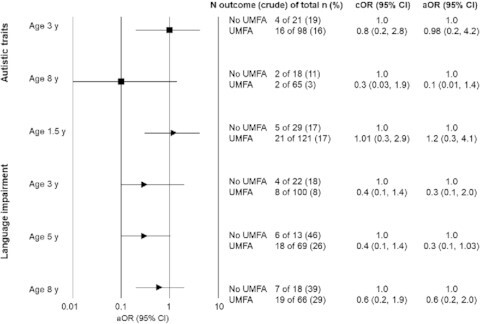

See corresponding editorial on page 1268.

## Introduction

Maintaining an adequate folate status before and during pregnancy is critical to prevent diseases caused by folate inadequacy, such as neural tube defects and megaloblastic anemia of pregnancy (1, 2). Women in most countries are recommended folic acid supplementation periconceptionally, and several countries have mandatory folic acid food fortification (1). Evidence of a more favorable neurodevelopmental outcome in the child after folic acid supplementation in pregnancy has recently emerged (1). At the same time, there has been increasing concern of potential adverse neurodevelopment in the children, caused by excess folic acid intake during pregnancy (2–7). Folic acid is the synthetic form of folate, a B vitamin required in one-carbon metabolism and thus essential for fetal brain development, including DNA and RNA biosynthesis and methylation (1). When folic acid intake is excessive, unmetabolized folic acid (UMFA) can accumulate in plasma (2, 4). Few studies have examined the effect of UMFA exposure in utero on offspring neurodevelopment. One study reported an association between high cord-blood UMFA concentrations and increased risk of autism spectrum disorder (ASD) in the United States (8).

Several commonly used types of antiseizure medication (ASM) interact with folate metabolism, causing low folate concentrations (9, 10). Prenatal ASM exposure is associated with increased risk of congenital anomalies and adverse neurodevelopment, such as increased risk of ASD and poor cognitive and verbal abilities (11, 12). Folate metabolism has been hypothesized as a target involved in the susceptibility to teratogenic effects of ASM (13–16). Women with epilepsy using ASM are consequently often recommended high-dose folic acid supplementation before and during pregnancy (17, 18). Several studies have found a protective effect of periconceptional folic acid supplementation on ASM-associated adverse neurodevelopment in children of women with epilepsy (19–23), but not on the risk of congenital malformations (24–26). In Norway, women with epilepsy using ASM are recommended a daily intake of 1–5 mg folic acid in the periconceptional period, depending on the ASM administered, and of 0.4 mg in the second and third trimesters. General agreement on the optimal dose of folic acid is lacking (12, 16, 27, 28). As women with epilepsy use both high-dose folic acid supplements during pregnancy and medication that interferes with folate metabolism, it is important to investigate the safety aspects of UMFA.

We have previously found a protective effect of periconceptional folic acid supplement use on ASM-associated risk of language impairment and autistic traits in young children (19–21). In the current study, we aimed to examine the association between pregnancy UMFA concentrations and risk of autistic traits and language impairment in children of women with epilepsy aged 1.5–8 y.

## Methods

### Study population

We included children of women with epilepsy using ASM and with analyzed plasma UMFA during pregnancy enrolled in The Norwegian Mother, Father and Child Cohort Study (MoBa). MoBa is an ongoing, prospective population-based pregnancy cohort study conducted by the Norwegian Institute of Public Health, and linked to the Medical Birth Registry of Norway (MBRN) (29). Participants were enrolled all over Norway from 1999 to 2008. The participation rate was 41% (29). The included women completed questionnaires in gestational weeks 17–19 and 30, and when the child was 1.5, 3, 5, and 8 y old. The questionnaires contained detailed information on social and medical background, medication use, vitamin supplement use during pregnancy, and child development after birth, including language abilities and autistic traits (29). Blood samples were obtained once during pregnancy between gestational weeks 17–19 and from the umbilical cord at birth (30). The current study is based on version 10 of the quality-assured MoBa data files.

### Epilepsy diagnosis and ASM use

We identified women with epilepsy from the self-reported MoBa questionnaires and from diagnostic data in the MBRN registered by the family physician or midwife. ASM use was reported in the MoBa questionnaires and in the MBRN. We have previously described the maternal epilepsy cohort in MoBa in detail (19–21, 31). The epilepsy diagnosis was validated with a retrospective validation survey sent out to 604 women with epilepsy (50% response rate), which included questions on epilepsy type, seizures during pregnancy, and folic acid dose (31). We have also validated the diagnosis and use of ASM by analysis of ASM concentrations in maternal blood and umbilical cord blood, and by diagnostic confirmation through medical record examination (*n* = 40) (31, 32). There was 100% agreement between the reported ASM in MoBa and ASM use registered in the hospital records, and 98% of the women in the retrospective survey confirmed the epilepsy diagnosis reported in MoBa (31, 32). Women with epilepsy in MoBa were representative of women with epilepsy in general in Norway (31).

### Folic acid supplement use

Women reported intake of folic acid supplement before and during pregnancy in the questionnaires at gestational weeks 17–19 and 30. They reported use in gestational week intervals, where zero represents the first day of the last menstrual period: −4 to 0, 0–4, 5–8, 9–12, and ≥13+ (first questionnaire) and 13–16, 17–20, 21–24, 25–28, and ≥29 (second questionnaire). The frequency of supplement intake was reported as daily, 4–6 times/wk, or 1–3 times/wk for each questionnaire. Plasma concentrations at gestational weeks 17–19 reflect folic acid supplement use during the first and second trimester (33, 34). We defined periconceptional folic acid supplement use as any intake during gestational weeks −4 to 12.

We collected data on the maternal folic acid supplement dose during pregnancy from the retrospective validation survey (31), as this information was not available in the MoBa questionnaires. A total of 97 of 227 children had available survey data. The women reported folic acid doses of 0.4 mg, 1–2 mg or ≥4 mg during gestational weeks −4 to 24. We grouped them into 2 groups according to the highest dose reported during this period: children exposed to low- or medium-dose folic acid (0.4–2 mg) and children exposed to high-dose folic acid (≥4 mg) use.

### Laboratory analyses

We analyzed folate and UMFA concentrations in maternal plasma samples from gestational weeks 17–19 at Bevital, Bergen, Norway (www.bevital.no). Samples were collected from singleton pregnancies of women with epilepsy using ASM in MoBa and with available samples in the MoBa biobank. Maternal plasma folate was defined as the sum of the concentrations of the 5-methyltetrahydrofolate (mTHF) and the mTHF-derived 4ɑ-hydroxy-5-methyltetrahydrofolate (hmTHF) metabolite (20, 35). mTHF is the biologically active folate form in plasma. It is unstable at room temperature but largely recovered as hmTHF (35, 36). The limits of quantification (LOQs) for mTHF, hmTHF, and UMFA were 0.13 nmol/L, 0.40 nmol/L, and 0.53 nmol/L, respectively (35). We dichotomized the UMFA concentrations into pregnancies with concentrations above and below the LOQ (37). Concentrations below the LOQ were reported as 0.0 nmol/L (37).

We analyzed plasma concentrations of valproate, carbamazepine, lamotrigine, levetiracetam, topiramate, and the oxcarbazepine monohydroxy-derivative metabolite in maternal samples from gestational weeks 17–19 and in umbilical cord blood at birth (20, 21, 31). For the statistical analyses, we calculated standardized ASM concentrations by normalizing the plasma concentrations relative to the range observed within each ASM group according to the following formula: 100 × (observed concentration − minimum concentration)/concentration range (20, 21, 38). For each child, the standardized ASM concentration was based on the mean of the maternal and the umbilical cord sample. If only 1 sample was present, this was used. For children exposed to ASM polytherapy, the sum of the mean of each standardized ASM concentration was given (20).

### Autistic traits

We examined autistic traits with the Social Communication Questionnaire (SCQ; **Supplemental Table 1**), as previously reported (19, 32). This validated, 40-item screening instrument was available in the MoBa questionnaires for ages 3 and 8 y (39–41). Different cutoffs regarding autistic traits have been defined from validation studies depending on age (39–41). We defined children to have autistic traits if the SCQ score was ≥11 points (39, 40).

### Language impairment

We examined language abilities at the different ages with 4 different, validated parent-reported language screening instruments (**Supplemental Table 2**), as reported previously (20, 21, 32): the Ages and Stages Questionnaires (ASQ) (42, 43), a 1-item question regarding expressive language (44), the Speech and Language Assessment Scale (SLAS) (45), and the Norwegian instrument Twenty Statements about Language-related Difficulties (Language 20) (46). Language impairment was based on the ASQ for age 1.5 y; the ASQ and the 1-item question on expressive language for age 3 y; the ASQ, the SLAS, and the Language 20 for age 5 y; and the semantic subscale of the Language 20 instrument for age 8 y (20, 21). Children with scores outside the cutoff in 1 or more of the language instruments at each age were defined as children with language impairment (20, 21).

### Covariates

The following covariates were included from the MoBa questionnaires and the MBRN (19–21): maternal age, smoking during pregnancy, maternal depression and anxiety during pregnancy [mean score >1.75 on the Hopkins Symptom Checklist at gestational weeks 17–19 (47)], and socioeconomic status (SES) measured as the sum of the following: low maternal education (≤9 y of schooling), low household income (total household income <60% of the national median in the child's birth year), and non-cohabiting mother.

### Statistical analysis

We used IBM SPSS Software version 25 (IBM Corporation) to perform the statistical analyses. For each outcome screening score, if missing answers were less than 29%, they were imputed using the estimation-maximization procedure in SPSS (19–21). We compared the UMFA concentrations in children with and without autistic traits or language impairment with the Mann-Whitney *U* test. We compared the number of children with and without autistic traits or language impairment stratified for UMFA quartile with chi-square test of independence or Fisher's exact test. The associations between plasma UMFA concentrations in pregnancy and language score and autistic trait score at ages 1.5–8 y were examined by using multiple linear regression models. We calculated the risk of language impairment or autistic traits at ages 1.5–8 y in children exposed to UMFA in pregnancy compared with those not exposed by using logistic regression models. All regression models were adjusted for standardized ASM concentrations and for each of the covariates described above if they changed the UMFA OR or B-coefficient by more than a change in the third decimal when examined separately with UMFA. We performed sensitivity analyses in order to separate the UMFA effect from the effect of folic acid supplement and ASM use:

1. We additionally adjusted for maternal folate concentration in the regression models. Although maternal UMFA and folate concentrations are strongly correlated (48, 49), the UMFA concentration varies across concentrations of folate, and UMFA can act independently of folate-mediated one-carbon metabolism (2).
2. We repeated the regression analyses after exclusion of periconceptional folic acid supplement nonusers, as we have previously found that nonuse of folic acid supplement in this period was associated with an increased risk of language impairment and autistic traits in the MoBa epilepsy cohort (19–21).
3. We compared the UMFA concentrations in children with and without autistic traits or language impairment in the following subgroups: ASM polytherapy, ASM monotherapy, and valproate, lamotrigine, and carbamazepine monotherapy, as the various ASMs may affect folate metabolism differently (9, 10, 48). We also examined the folic acid high-dose and low- or medium-dose groups separately, as the concentrations of UMFA may vary between individuals within the same dose category (48, 49).

Two-sided *P* values <0.05 were considered statistically significant.

### Ethical approval and informed consent

All data and material in MoBa are collected with informed consent from the participants. The establishment of MoBa and initial data collection was based on a license from the Norwegian Data Protection Agency and approval from the Regional Committees for Medical and Health Research Ethics. The MoBa cohort is now based on regulations related to the Norwegian Health Registry Act. The current study was approved by the Regional Committees for Medical and Health Research Ethics (reference number 2011/1616).

## Results

### Characteristics

**FIGURE 1:**
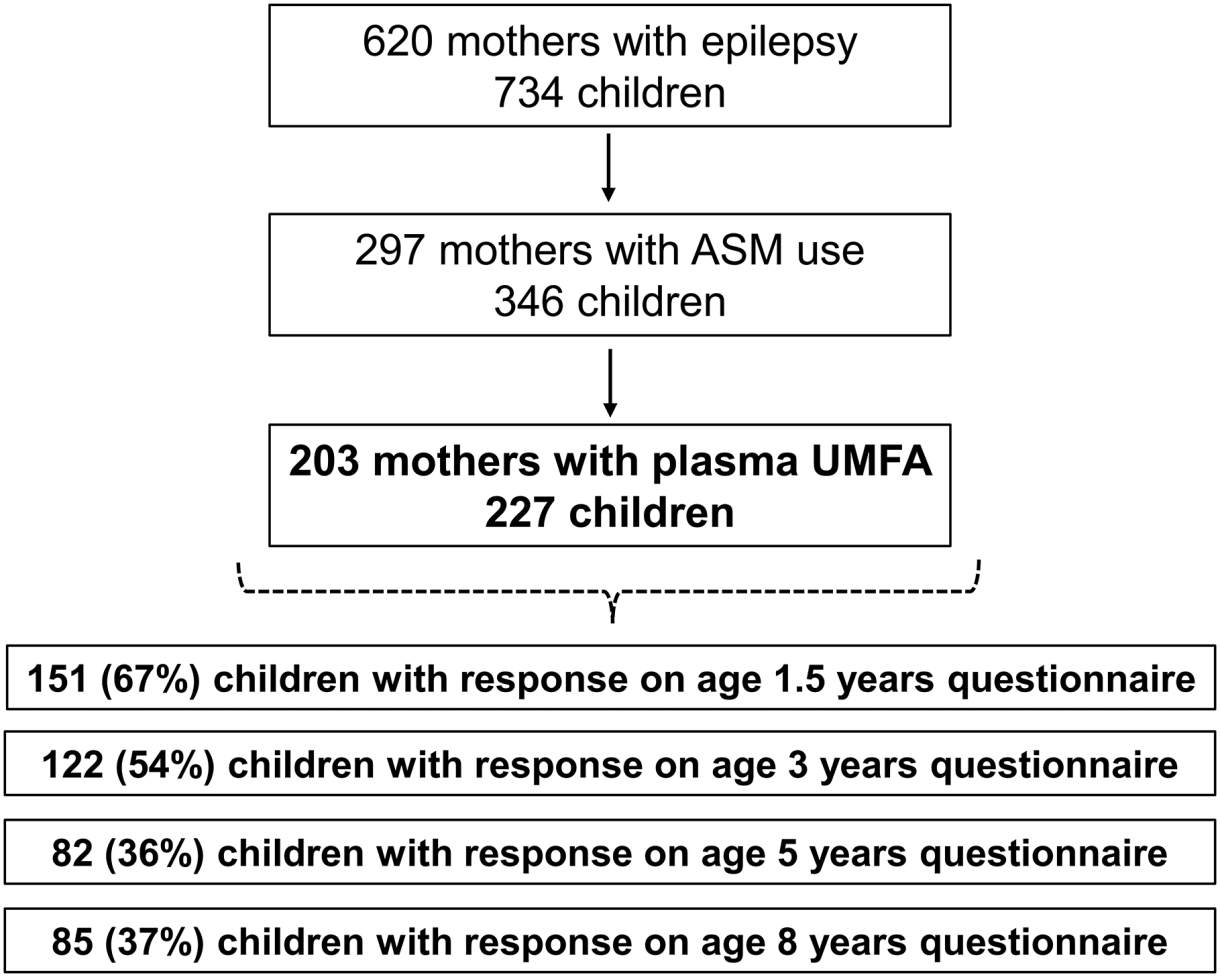
Flowchart of included and excluded cases. ASM, antiseizure medication; UMFA, unmetabolized folic acid.

We included 227 children of 203 women with epilepsy (**Figure 1**). We identified 183 children (81%) exposed to ASM monotherapy and 44 children (19%) exposed to ASM polytherapy (**Table 1**). In 208 (92%) children, the mothers reported use of folic acid supplements at any time between gestational week −4 and 20 (Table 1). Maternal plasma UMFA concentrations were detected during pregnancy for 177 children (Table 1). Among the 97 children with data from the retrospective survey, 76 children had precise information on folic acid dose (Table 1).

**TABLE 1:** Characteristics of children of mothers with epilepsy stratified for detected UMFA concentration during pregnancy^1^

	Children of mothers without UMFA (*n* = 50)	Children of mothers with UMFA (*n* = 177)
Maternal age, median (range), y	29.0 (18.0)	29.0 (23.0)
Smoking in pregnancy, *n* (%)		
No	40 (80)	148 (84)
Yes	10 (20)	28 (16)
Missing	0 (0)	1 (1)
Maternal depression/anxiety symptoms,^2^*n* (%)		
No	36 (72)	142 (80)
Yes	9 (18)	25 (14)
Missing	5 (10)	10 (6)
SES (low education or low household income or non-cohabiting mother),^3^*n* (%)		
No, neither	40 (80)	142 (80)
Yes, 1 or more	9 (18)	29 (16)
Missing	1 (2)	6 (3)
ASM polytherapy use, *n* (%)		
No	44 (88)	139 (79)
Yes	6 (12)	38 (21)
Any valproate use, *n* (%)	10 (20)	33 (19)
Any carbamazepine use, *n* (%)	14 (28)	54 (31)
Any lamotrigine use, *n* (%)	19 (38)	76 (43)
Any levetiracetam use, *n* (%)	6 (12)	19 (11)
Any topiramate and any oxcarbazepine use, *n* (%)	5 (10)	25 (14)
ASM concentrations (µmol/L),^4^ median (range)	33.1 (98)	40.9 (170)
Missing, *n* (%)	8 (16)	9 (5)
Any folic acid supplement use gestational weeks −4 to 20, *n* (%)		
No	7 (14)	6 (3)
Yes	42 (84)	166 (94)
Missing	1 (2)	5 (3)
Periconceptional folic acid supplement use,^5^*n* (%)		
No	10 (20)	28 (16)
Yes	39 (78)	143 (81)
Missing	1 (2)	6 (3)
Folic acid dose gestational weeks −4 to 24, *n* (%)		
Low or medium dose (0.4–2 mg)	10 (20)	27 (15)
High dose (≥4 mg)	6 (12)	33 (19)
Missing	34 (68)	117 (66)
UMFA, median (range), nmol/L	0.0 (0.0)	1.8 (302)

1 ASM, antiseizure medication; SES, socioeconomic status; UMFA, unmetabolized folic acid.

2 Mean score >1.75 on the Hopkins Symptom Checklist in gestational weeks 17–19.

3 SES measured as the sum of the following: low maternal education (≤9 y of schooling), low total household income (<60% of the national median in the child's birth year), or non-cohabiting mother.

4 Based on standardized ASM concentrations from maternal plasma samples in gestational weeks 17–19 and umbilical cord blood (see text).

5 Any use of folic acid supplement in the period from gestational weeks −4 to 12 (gestational week 0 starts with the first day of the last menstrual period).

### Plasma UMFA concentration and association with autistic traits

**FIGURE 2:**
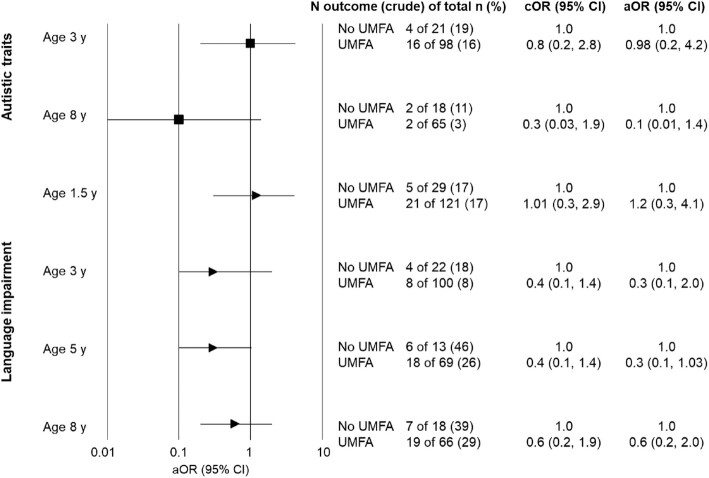
The aOR of autistic traits or language impairment in children aged 1.5–8 y with exposure to plasma UMFA during pregnancy compared with no exposure. For covariates in the adjusted model, see the text. aOR, adjusted OR; cOR, crude OR; UMFA, unmetabolized folic acid.

Median UMFA concentrations did not differ between children with and without autistic traits at age 3 and 8 y (**Table 2**). The proportion of children with autistic traits in the highest UMFA concentration quartile did not differ from the proportion in each of the lower UMFA quartiles (Table 2). In the adjusted multiple linear regression analysis, we found no statistically significant associations between UMFA concentrations and SCQ scores at ages 3 and 8 y (**Table 3**). High UMFA concentrations were associated with low SCQ scores and fewer autistic traits at age 3 y (unstandardized B: −0.01; 95% CI: −0.03, 0.004; *P* = 0.14). For age 8 y, the association was in the opposite direction (B: 0.01; 95% CI: −0.02, 0.03; *P* = 0.61) (Table 3). Children exposed to UMFA had no increased risk of autistic traits compared with children not exposed in both age groups (**Figure 2**).

**TABLE 2:** Median UMFA concentration (nmol/L) in children with and without autistic traits or language impairment at age 1.5, 3, 5, and 8 y, and the number of children in UMFA concentration quartile 1 (lowest) to 4 (highest)^1^

	Autistic traits^2^				Language impairment^3^							
	3 y		8 y		1.5 y		3 y		5 y		8 y	
	Yes (*n* = 20)	No (*n* = 99)	Yes (*n* = 4)	No (*n* = 79)	Yes (*n* = 26)	No (*n* = 124)	Yes (*n* = 12)	No (*n* = 110)	Yes (*n* = 24)	No (*n* = 58)	Yes (*n* = 26)	No (*n* = 58)
UMFA, *n* (%)												
First quartile	4 (20)	18 (18)	2 (50)	18 (23)	5 (19)	27 (22)	4 (33)	19 (17)	6 (25)	10 (17)	7 (27)	13 (22)
Second quartile	4 (20)	24 (24)	0 (0)	19 (24)	8 (31)	27 (22)	2 (17)	26 (24)	5 (21)	15 (26)	3 (12)	16 (28)
Third quartile;	6 (30)	26 (26)	1 (25)	17 (22)	7 (27)	36 (29)	4 (33)	29 (26)	5 (21)	16 (28)	8 (31)	11 (19)
Fourth quartile	6 (30)	31 (31)	1 (25)	25 (32)	6 (23)	34 (27)	2 (17)	36 (33)	8 (33)	17 (29)	8 (31)	18 (31)
UMFA, median (range), nmol/L	1.3 (75)	1.5 (303)	0.9 (91)	1.3 (169)	1.2 (59)	1.3 (303)	1.2 (17)	1.5 (303)	1.6 (44)	1.7 (182)	1.7 (91)	1.2 (169)

1 There were no differences in UMFA concentrations between children with and without autistic traits or language impairment for all ages (Mann-Whitney *U* test). There were no differences in number of children with autistic traits or language impairment in the highest UMFA quartile (fourth quartile) compared with each of the lower quartiles for all ages (chi-square test of independence or Fisher's exact test). Quartiles based on UMFA concentration (nmol/L): first quartile. UMFA <0.56 nmol/L (*n* = 55); second quartile, UMFA ≥0.56 nmol/L and <1.16 nmol/L (*n* = 57); third quartile, UMFA ≥1.16 nmol/L and < 3.99 nmol/L(*n* = 58); fourth quartile, UMFA ≥3.99 nmol/L (*n* = 57). ASQ, Ages and Stages Questionnaires; Language 20, Twenty Statements about Language-related Difficulties; SCQ, Social Communication Questionnaire; SLAS, Speech and Language Assessment Scale; UMFA, unmetabolized folic acid.

2 Autistic traits at ages 3 and 8 y according to the SCQ. At age 3 y, 3 children and at age 8 y, 2 children were excluded due to too many missing answers.

3 Language impairment at age 1.5 y according to the ASQ; at age 3 y according to the ASQ and 1 question on expressive language delay; at age 5 y according to the ASQ, Language 20, and the SLAS; and at age 8 y according to the Language 20. At age 1.5 y, 1 child and at age 8 y, 1 child were excluded due to too many missing answers.

**TABLE 3:** Association between the maternal concentration of UMFA (nmol/L) in gestational weeks 17–19 and autistic trait scores and language scores at age 1.5, 3, 5, and 8 y^1^

	Score interpretation	UMFA, unstandardized β	95% CI	SE, unstandardized β	UMFA, standardized β	*P*
Autistic traits score^2^						
3 y						
SCQ (*n* = 119)	Lower is normal	−0.01	−0.03, 0.004	0.01	−0.14	0.14
8 y						
SCQ (*n* = 83)	Lower is normal	0.01	−0.02, 0.03	0.01	0.06	0.61
Language scores^3^						
1.5 y						
ASQ (*n* = 150)	Higher is normal	0.02	−0.02, 0.05	0.02	0.08	0.36
3 y						
EL (*n* = 121)	Higher is normal	0.001	−0.003, 0.005	0.002	0.05	0.60
ASQ (*n* = 121)	Higher is normal	0.01	−0.02, 0.04	0.01	0.07	0.48
5 y						
ASQ (*n* = 82)	Higher is normal	0.003	−0.03, 0.04	0.02	0.02	0.85
SLAS (*n* = 81)	Higher is normal	0.000	−0.004, 0.003	0.002	−0.02	0.87
Lang 20 (*n* = 82)	Lower is normal	−0.02	−0.07, 0.04	0.03	−0.07	0.57
8 y						
Lang 20 (*n* = 84)	Lower is normal	−0.01	−0.04, 0.02	0.02	−0.08	0.50

1 The associations between UMFA and autistic trait score and between UMFA and language score, respectively, were examined separately for each age and score by using multiple linear regression. Variables in the adjusted model: all models were adjusted for standardized ASM concentrations (see text). In addition, the following covariates were selected one by one and included in the model if the UMFA standardized β changed with more than a change in the third decimal: maternal age, SES (non-cohabiting mother or low education or low household income), symptoms of anxiety and/or depression during pregnancy (mean score >1.75 on the Hopkins Symptom Checklist in gestational weeks 17–19) and smoking in pregnancy. ASQ, Ages and Stages Questionnaires; EL, expressive language skills score; Lang 20, Twenty Statements about Language-related Difficulties (Language 20); SCQ, Social Communication Questionnaire; SES, socioeconomic status; SLAS, Speech and Language Assessment Scale; UMFA, unmetabolized folic acid.

2 At age 3 y, 3 children and at age 8 y, 2 children were excluded due to too many missing answers.

3 At age 1.5 y, 1 child was excluded due to too many missing answers (ASQ score). At age 3 y, 1 child was excluded in the EL score due to too many missing answers, and another child was excluded in the ASQ score for the same reason. At age 5 y, 1 child was excluded due to missing answers on the SLAS score, and at age 8 y 1 child was excluded due to too many missing answers on the Language 20 Semantic subscale.

### Plasma UMFA concentration and association with language impairment

Median UMFA concentrations did not differ between children with and children without language impairment for all age groups (Table 2). The proportion of children with language impairment in the highest UMFA concentration quartile did not differ from the proportion in each of the lower UMFA quartiles for all ages (Table 2). Adjusted multiple linear regression analyses found no statistically significant associations between UMFA concentrations and language scores (Table 3). The direction of association indicated that high UMFA concentrations were rather associated with less language impairment for all scores, except for SLAS at age 5 y (Table 3). There was no statistically significant increased risk of language impairment in children exposed to UMFA compared with those not exposed for all age groups (Figure 2).

### Sensitivity analyses

Additional adjustment for maternal folate concentration did not change the lack of significant associations between pregnancy UMFA concentrations and risk of autistic traits or language impairment (data not shown); neither did removal of the children where the mothers reported no periconceptional folic acid supplement use from the analyses (data not shown). Subgroup analyses for ASM monotherapy, valproate monotherapy, lamotrigine monotherapy, and ASM polytherapy revealed no statistically significant difference in median UMFA concentrations in children with and without autistic traits or language impairment (data not shown). In the carbamazepine monotherapy group (*n* = 19), the median UMFA concentration was significantly higher in children without autistic traits at age 8 y compared with children with autistic traits [median of 2.79 (range 75) nmol/L compared with median of 0.0 (range: 0) nmol/L]. Subgroup analyses in children exposed to high-dose (*n* = 39) and in children exposed to low- or medium-dose (n = 37) folic acid supplements revealed no statistically significant difference in median UMFA concentrations in children with and without autistic traits or language impairment (data not shown).

## Discussion

In this study, we found no statistically significant association between plasma UMFA concentrations during pregnancy and autistic traits or language impairment in young and school-aged ASM-exposed children of women with epilepsy. In children exposed to UMFA during pregnancy compared with unexposed children, there was no increased risk of autistic traits or language impairment at any age.

No adverse associations between UMFA concentrations and autistic traits or language impairment in our data persisted after adjustment for maternal folate concentrations. High UMFA concentrations are related to folic acid supplement dose and frequency of intake (2, 4), and strongly correlated with maternal folate concentrations (48, 49). Hence, we cannot completely separate a potentially negative effect of UMFA from the positive effect of folic acid supplement use on autistic traits and language impairment (19–21). However, UMFA may act through pathways not normally associated with folate (2). Therefore, we adjusted for folate concentrations and we also excluded children of women who had reported no periconceptional folic acid supplement use from the sensitivity analyses. This did not change our results. None of the screening instrument scores showed any evidence of high UMFA concentrations being linked to autistic traits or language impairment.

Previous studies examining the association between UMFA during pregnancy and neurodevelopmental outcomes in children of women with epilepsy are lacking (12, 13). In the general population, 1 study found an association between high UMFA concentrations in cord blood and increased risk of ASD in some population groups in the United States (8). Although detected in cord blood, other studies found that UMFA was unlikely to accumulate in the fetus, both after high and low doses of supplement and with different fortification guidelines (37, 49, 50). Considering the interaction between ASM and folate metabolism (9, 10), and the need for higher folic acid doses in ASM-treated women, findings from the general population should not automatically be generalized to women with epilepsy. Norway does not have mandatory folic acid food fortification, and our findings may not be representative for countries with such fortification.

In the sensitivity analyses, we found no statistically significant difference in UMFA concentrations between children with and without autistic traits or language impairment when examining groups with similar folic acid exposure in utero (≥4 mg or 0.4–2 mg, respectively). Two reports from the Neurodevelopmental Effects of Antiepileptic Drugs (NEAD) study of ASM-exposed children aged 3 and 6 y of mothers with epilepsy on folic acid dose and IQ found that self-reported periconceptional folic acid supplementation >0.4 mg/d was associated with better cognitive development and verbal abilities compared with non–supplement users (22, 23). These studies did not measure UMFA concentrations, but the majority of women used doses >0.4 mg and thus UMFA would probably be present (49). One study from the general population found a U-shaped increased risk of ASD after low-dose and high-dose (>5 mg) folic acid supplementation during pregnancy (6). Another study from the general population found lower psychomotor development and verbal abilities in children aged 1 y after exposure to folic acid doses >5 mg (7). Data from the general population on high-dose folic acid use in pregnancy and adverse neurodevelopment in the children are still regarded as inconclusive (2–5).

Strengths of our study include a validated epilepsy diagnosis; measurements of UMFA, folate, and ASM during pregnancy; and prospectively collected data on language impairment and autistic traits up to age 8 y. We have adjusted for relevant confounders and the effect of folate and ASM. None of the children were assessed by a neuropsychologist, but our screening instruments are sensitive and have been validated (39–46). Parents are considered good evaluators of the language abilities of their children (51). Limitations of the study include blood samples collected only once during pregnancy. The UMFA concentration depends on a variety of factors, which, in addition to individual kinetic properties of UMFA and folate metabolism, also include the time gap between folic acid supplement intake and sample collection (2). As the latter is unknown in MoBa, and we cannot exclude UMFA exposure earlier or later in the pregnancy with the MoBa design, misclassification of the UMFA exposure in some individuals cannot be ruled out. There is some loss to follow-up in MoBa, particularly at age 5 and 8 y. We have previously reported that children with language impairment at ages 1.5–3 y and with no exposure to periconceptional folic acid supplement use were less likely to respond to the 5- and 8-y questionnaire (21). We cannot rule out that this may introduce selection bias affecting our results, considering the strong correlation between UMFA and maternal folate (48). However, we examined exposure–outcome associations, with the UMFA exposure being unknown to the MoBa participants, and the outcome data collected prospectively months and years after the exposure. We thus believe that it is less likely that our results are affected by selection bias. Other limitations include retrospectively collected folic acid dose data available for only a subgroup of women. White children and children of mothers with high SES and education are overrepresented in MoBa (52), which may limit the external generalizability of our findings.

In conclusion, we found no association between maternal UMFA concentrations during pregnancy and risk of autistic traits and language impairment in ASM-exposed children of women with epilepsy. Our findings do not support an association between UMFA in pregnancy and adverse neurodevelopment in children of women with epilepsy.

## Supplementary Material

nqab436_Supplemental_File

## Data Availability

The consent given by the participants does not allow for storage of data on an individual level in repositories or journals. Researchers who want access to data sets for replication should apply to MoBa (e-mail: datatilgang@fhi.no). Access to data sets requires approval from the Regional Committee for Medical and Health Research Ethics in Norway and an agreement with MoBa.

## Notes

Supported by the Norwegian Epilepsy Foundation, Norwegian Chapter International League Against Epilepsy, Det alminnelige medisinske forskningsfond ved Universitetet i Bergen, Dr. Nils Henrichsen og hustru Anna Henrichsens legat, and Advokat Rolf Sandberg Reberg og Ellen Marie Rebergs Legat til Epilepsiforskning. The sources of funding had no role in the design of the study or the interpretation of the results. The Norwegian Mother, Father and Child Cohort Study is supported by the Norwegian Ministry of Health and Care Services and the Ministry of Education and Research.

Supplemental Tables 1 and 2 are available from the “Supplementary data” link in the online posting of the article and from the same link in the online table of contents at https://academic.oup.com/ajcn/.

Abbreviations used: aOR, adjusted OR; ASD, autism spectrum disorder; ASM, antiseizure medication; ASQ, Ages and Stages Questionnaires; hmTHF, 4ɑ-hydroxy-5-methyltetrahydrofolate; LOQ, limit of quantification; MBRN, Medical Birth Registry of Norway; MoBa, Norwegian Mother, Father, and Child Cohort Study; mTHF, 5-methyltetrahydrofolate; Language 20, Twenty Statements about Language-related Difficulties; SCQ, Social Communication Questionnaire; SES, socioeconomic status; SLAS, Speech and Language Assessment Scale; UMFA, unmetabolized folic acid.
