# Supplementary material for: Plasma unmetabolized folic acid in pregnancy and risk of autistic traits and language impairment in antiseizure medication–exposed children of women with epilepsy
Source: Am J Clin Nutr. 2022 Jan 5;115(5):1432–40. doi: 10.1093/ajcn/nqab436 (PMC9071448; doi:10.1093/ajcn/nqab436)
Supplement: nqab436_Supplemental_File [file nqab436_supplemental_file.pdf]

Plasma unmetabolized folic acid in pregnancy and risk of autistic traits and language impairment in antiseizure medication-exposed children of women with epilepsy

Online Supplementary Material

Contents

Supplementary Table 1. The Social Communication Questionnaire (SCQ) 2

Supplementary Table 2. Language impairment screening instruments 4

Supplementary Table 1. The Social Communication Questionnaire (SCQ)

The 40-item screening instrument The Social Communication Questionnaire (SCQ) from the questionnaire at ages 3 and 8 years in the Norwegian Mother, Father and Child Cohort Study (MoBa).

**The Social Communication Questionnaire (SCQ)**<br>*Mark one box per question, whether you think it applies for your child or not. Response options: 1-“Yes”, 2-“No”.*
Your child’s use of language with others
1. Is he/she now able to talk using short phrases or sentences?
2. Do you have a to and fro "conversation" with her/him that involves taking turns or building on what you have said?
3. Does she/he ever use odd phrases or say the same thing over and over in almost exactly the same way (either phrases that she/he hears other people use or ones that she/he makes up)?
4. Does your child ever use socially inappropriate questions or statements? For example, does your child ever regularly ask personal questions or make personal comments at awkward times?
5. Does your child ever get his/her pronouns mixed up (e.g., saying you or he/she for I)?
6. Does your child ever use words that he/she seems to have invented or made up her/himself; put things in odd, indirect ways; or use metaphorical ways of saying things (e.g., saying hot rain for steam)?
7. Does your child ever say the same thing over and over in exactly the same way or insist that you say the same thing over and over again?
About behavior and specific things that children can think of doing
8. Does your child ever have things that he/she seems to have to do in a very particular way or order or rituals that the child insists that you go through?
9. Does your child’s facial expression usually seem appropriate to the particular situation, as far as you can tell?
10. Does your child ever use your hand like a tool or as if it were part of his/her own body (e.g., pointing with your finger or putting your hand on a doorknob to get you to open the door)?
11. Does your child ever have any interests that preoccupy him/her and might seem odd to other people (e.g., traffic lights, drainpipes, or timetables)?
12. Does your child ever seem to be more interested in parts of a toy or an object (e.g., spinning the wheels of a car), rather than in using the object as it was intended?
13. Does your child ever have any special interests that are unusual in their intensity, but otherwise appropriate for his/her age and peer group (e.g., trains or dinosaurs)?
14. Does your child ever seem to be unusually interested in the sight, feel, sound, taste, or smell of things or people?
15. Does your child ever have any mannerisms or odd ways of moving his/her hands or fingers, such as flapping or moving his/her fingers in front of his/her eyes?
16. Does your child ever have any complicated movements of his/her whole body, such as spinning or repeatedly bouncing up and down?
17. Does your child ever injure himself/herself deliberately, such as by biting his/her arm or banging his/her head?
18. Does your child ever have any objects (other than a soft toy or comfort blanket) that he/she has to carry around?
About social development and interest in others
19. Does your child have any particular friends or a best friend?
20. Does your child ever talk with you just to be friendly (rather than to get something)?
21. Does your child ever spontaneously copy you (or other people) or what you are doing (such as vacuuming, gardening, or mending things)?

22. Does your child ever spontaneously point at things around him/her just to show you things (not because he/she wants them)?
23. Does your child ever use gestures, other than pointing or pulling your hand, to let you know what he/she wants?
24. Does your child nod his/her head to indicate yes?
25. Does your child shake his/her head to indicate no?
26. Does your child usually look at you directly in the face when doing things with you or talking with you?
27. Does your child smile back if someone smiles at him/her?
28. Does your child ever show you things that interest him/her to engage your attention?
29. Does your child ever offer to share things other than food with you?
30. Does your child ever seem to want you to join in his/her enjoyment of something?
31. Does your child ever try to comfort you when you are sad or hurt?
32. If your child wants something or wants help, does he/she look at you and use gestures with sounds or words to get your attention?
33. Does your child show a normal range of facial expressions?
34. Does your child ever spontaneously join in and try to copy the actions in social games, such as The Mulberry Bush or London Bridge Is Falling Down?
35. Does your child play any pretend or make-believe games?
36. Does your child seem interested in other children of approximately the same age whom he/she does not know?
37. Does your child respond positively when another child approaches him/her?
38. If you come into a room and start talking to your child without calling his/her name, does he/she usually look up and pay attention to you?
39. Does your child ever play imaginative games with another child in such a way that you can tell that each child understands what the other is pretending?
40. Does your child play cooperatively in games that need some form of joining in with a group of other children, such as hide-and-seek or ball games?

Supplementary Table 2. Language impairment screening instruments

Overview of language impairment screening instruments in the Norwegian Mother, Father and Child Cohort Study (MoBa) questionnaires. The table includes the Ages and Stages Questionnaires (ASQ) communication scale (ages 1.5, 3 and 5 years), a one-item question on expressive language skills (age 3 years), the 13-item version of the Speech and Language Assessment Scale (SLAS) (age 5 years), and the Twenty Statements about Language-related Difficulties (Language 20) (complete instrument at age 5 years, at age 8 years only the semantic subscale was included in the questionnaire).

**The Ages and Stages Questionnaires (ASQ) communication scale**<br>*The child's ability to talk and understand. Response options: "yes", "sometimes", "not yet"*
1.5 years
1. When you ask him/her, does your child go into another room to find a familiar toy or object? (You might ask, "Where is your ball?", or say, "Bring me your coat" or "Go get your blanket").
2. Does your child say eight or more words in addition to "mama" and "Dada"?
3. Without showing him/her first, does your child point to the correct picture when you say, "Show me the kitty" or ask "Where is the dog"?
3 years
1. Without showing him/her first, does your child point to the correct picture when you say, "Where is the cat" or "Where is the dog"? Your child must only point at the correct picture
2. When you ask your child to point to his/her eyes, nose, hair, feet, ears, and so forth, does he/she correctly point to at least seven body parts? (The child can point to parts of himself/herself, you, or a doll.)
3. Does your child make sentences that are three or four words long?
4. Without giving him/her help by pointing or using gestures, ask your child to "Put the shoe on the table" and "Put the book under the chair". Does your child carry out both of these directions correctly?
5. When looking at a picture book, does your child tell you what is happening or what action is taking place in the picture? (For example, "Barking", "Running", "Eating" and "Crying"?) You may ask, "What is the dog (or boy) doing?"
6. Can your child tell you at least two things about an object he/she is familiar with? If you say, for example, "Tell me about your ball", will your child answer by saying something like "It is round, I can throw it, it is big"?
5 years
1. Can your child tell you at least two things about common object? For example, if you say to your child, "Tell me about the ball", does he say something like, "It is round. I throw it. It is big"?
2. Without giving your child help by pointing or repeating directions, does your child follow three directions that are unrelated to one another? Give all three directions before your child starts. For example, you may ask your child to "Clap your hands, walk to the door and sit down" or "Give me the pen, open the book and stand up".
3. Does your child use four- and five- word sentences? For example, does your child say, "I want the car"?
4. When talking about something that already happened, does your child use words that end in "ed" such as walked, jumped or played? Ask your child questions, such as "How did you get to the store?" "What did you do at your friend's house?" ("We played").

5. Does your child use comparison words, such as heavier, stronger or shorter? Ask your child questions, such as "A car is big, but a bus is _____" (bigger); "A cat is heavy, but a man is ____" (heavier); A TV is small, but a book is ____ " (smaller).
6. Does your child answer the following questions: 1) "What do you do when you are hungry?" (Acceptable answers include: "Get food", "Eat", "Ask for something to eat", and "Have a snack".) 2) "What do you do when you are tired?" (Acceptable answers include: "Take a nap", "Rest", "Go to sleep", "Go to bed", "Lie down", and "Sit down.")
7. Does your child repeat the sentences shown below back to you, without any mistakes? You may repeat each sentence one time. Mark "yes" if your child repeats both sentences without mistakes or "sometimes" if your child repeats one sentence without mistakes. "Jane hides her shoes for Maria to find." "Al read the blue book under his bed."
**1-item question on expressive language skills**<br>*About your child's language skills (enter a cross for the option which best describes the way your child talks.)*
1 = Not yet talking
2 = He/she is talking, but you can't understand him/her
3 = Talking in one word-utterances, such as "milk" or "down"
4 = Talking in 2- or 3-word phrases, such as "me got ball" or "give doll"
5 = Talking in fairly complete sentences, such as 'I got a doll' or 'can I go outside?'
6 = Talking in long and complicate sentences, such as 'when I went to the park, I went on the swings' or 'I saw a man standing on the corner'
**The Speech and Language Assessment Scale (SLAS)**<br>*About the child's abilities and skills compared with peers. Enter a cross from 1-5 for each line according to how well the statement fits your child. Response options: 1-"very much lower", 2-2, 3-"typical for age", 4-4, 5-"very much higher"*
1. My child's ability to ask questions properly is…
2. My child's ability to answer questions properly is…
3. My child's ability to say sentences clearly enough to be understood by strangers is…
4. The number of words my child knows is…
5. My child's ability to use his/her words correctly is…
6. My child's ability to get his/her message across to others when talking is…
7. My child's ability to use proper words when talking to others is…
8. My child's ability to get what he/she wants by talking is…
9. My child's ability to start a conversation going with other children is…
10. My child's ability to keep a conversation going with other children is…
11. The length of this child's sentences is…
12. My child's ability to make 'grown up' sentences is…
13. My child's ability to correctly say the sounds in individual words is…
**Twenty Statements about Language-related Difficulties (Language 20)**<br>*How do these statements fit the child? Response options: 1-"doesn't fit the child, absolutely wrong", 2-2, 3-"both yes and no", 4-4, 5-"fits well with the child, absolutely right"*
Semantic subscale
1. Forgets words s/he knows the meaning of
2. Confuses words with similar meaning (e.g. shirt, sweater, jacket)
3. Has difficulty understanding the meaning of common words
4. Has difficulty answering questions as quickly as other children
5. Is often searching for the right words
6. Uses incomplete sentences
7. Uses short sentences when s/he answers questions
8. Has difficulty retelling a story s/he has heard
Receptive subscale

9. It doesn't seem like what s/he is learning is remembered
10. Has difficulty remembering things
11. Has difficulty understanding what others are saying
12. Misconceive instructions and messages
13. Has problems remembering messages
14. Misunderstands context and what is going on
Expressive subscale
15. Is difficult to understand
16. Has difficulty expressing wishes and needs
17. Is not understood by others
18. Seldom initiates conversation with others
19. Has difficulties in pronunciation
20. Is not able to have a dialogue with peers
